# miR-200 affects tamoxifen resistance in breast cancer cells through regulation of MYB

**DOI:** 10.1038/s41598-019-54289-6

**Published:** 2019-12-11

**Authors:** Yu Gao, Wenzhi Zhang, Chengwen Liu, Guanghua Li

**Affiliations:** 1 Department of General Surgery, Tianjin First Central Hospital, No.24, Fukang Road, Nankai District, Tianjin 300204 China; 2 Innoscience Research Sdn Bhd, Suites B-5-7, Level 5, Sky Park @ One City, Jalan USJ 25/1, 47650 Subang Jaya, Selangor Malaysia; 3 Department of Obstetrics and Gynecology, Maternity and Child Health Care of Zaozhuang, Zaozhuang, 277100 Shandong province China; 4 Department of General Surgery, The Second Hospital of Shandong University, No.247 Beiyuan Road, Tianqiao District, Jinan City, Shandong Province 250033 China

**Keywords:** Breast cancer, Breast cancer

## Abstract

Resistance to tamoxifen is a major clinical challenge. Research in recent years has identified epigenetic changes as mediated by dysregulated miRNAs that can possibly play a role in resistance to tamoxifen in breast cancer patients expressing estrogen receptor (ER). We report here elevated levels of EMT markers (vimentin and ZEB1/2) and reduced levels of EMT-regulating miR-200 (miR-200b and miR-200c) in ER-positive breast cancer cells, MCF-7, that were resistant to tamoxifen, in contrast with the naïve parental MCF-7 cells that were sensitive to tamoxifen. Further, we established regulation of c-MYB by miR-200 in our experimental model. C-MYB was up-regulated in tamoxifen resistant cells and its silencing significantly decreased resistance to tamoxifen and the EMT markers. Forced over-expression of miR-200b/c reduced c-MYB whereas reduced expression of miR-200b/c resulted in increased c-MYB We further confirmed the results in other ER-positive breast cancer cells T47D cells where forced over-expression of c-MYB resulted in induction of EMT and significantly increased resistance to tamoxifen. Thus, we identify a novel mechanism of tamoxifen resistance in breast tumor microenvironment that involves miR-200-MYB signaling.

## Introduction

Breast cancer is a major problem in China and world over. Its now the most common cancer among Chinese women^1^ and, by some estimates, accounts for 12.2% of all newly diagnosed breast cancers and 9.6% of all breast cancer deaths worldwide^2^. The numbers are on increase more so because of the increasing awareness^3^. This calls for improvements in the therapeutic options for the better clinical outcomes. Breast cancer has many subtypes^4^. Our team’s interest has been focused on the Estrogen Receptor (ER)-positive breast cancers that have turned refractory to the targeted therapy. Tamoxifen is the standard therapy for breast cancers that are ER-positive^5^, however, its well known that a majority of patients develop resistance against it. A number of mechanisms have been proposed and explored for the basis of acquired tamoxifen resistance of ER-positive breast cancer^6^, but the clinical problem continues to exist.

Among the many explored mechanisms, the process of epithelial-mesenchymal-transition (EMT), has been linked to acquired tamoxifen resistance in many studies^7,8^. Therefore, in the present study wherein we generated a tamoxifen-resistant subline of the well characterized ER-positive MCF-7 breast cancer cells, we started with an evaluation of induction of EMT. We also focused on the role of miRNAs, particularly the miR-200 family of miRNAs because miR-200 family is a well-known regulator of EMT^9,10^. Even though the role of miR-200s in anti-estrogen therapy of breast cancer has been suggested^11^, the mechanism remains unexplored. In particular, the possible induction of EMT in a tamoxifen resistance mechanism that also includes miR-200s remains unknown. Further, there is no information on the gene targets of miR-200s that can functionally mediate the phenotype of tamoxifen resistance. There is lack of any knowledge on the topic. In addition, in recent years, non-coding RNAs, a class to which miRNAs also belong, have been linked with the phenomenon of tamoxifen resistance in breast cancer^12,13^. With this background information, we carefully planned our present study the role of EMT and miR-200s in acquired tamoxifen resistance of ER-positive breast cancer cells. We also designed our study to elucidate a novel target of miR-200s, the oncogenic c-MYB, that is mechanistically involved in induction of EMT and resulting tamoxifen resistance.

## Materials and Methods

### Cell culture and other reagents

MCF-7 and T47D cells were obtained from Beijing Zhongyuan Limited (Beijing, China). Tamoxifen resistant MCF-7 cells, TAM-MCF7 were developed in our own laboratory by dose escalation method wherein we treated MCF-7 cells with increasing doses of tamoxifen for over 4 months. Cells were cultured in 5% CO_2_ humidified atmosphere at 37 °C. Tamoxifen was obtained from Sigma Chemical Company (Shanghai, China). Real time RT-PCR Primers for c-MYB and GAPDH were same as reported elsewhere^14^.

### MTS assay

MTS (3-(4,5-dimethylthiazol-2-yl)-5-(3-carboxymethoxyphenyl)-2-(4-sulfophenyl)-2H-tetrazolium) assay was employed to assess cell proliferation. Cell were first seeded overnight before the start of any experiment. Adequate replicates were taken and absorbance was measured on a spectrophotometer.

### Quantitative RT-PCR

We used mirVana RNA isolation kit to extract RNA. Realtime RT-PCR was performed as per the conditions described earlier^14^ on a BioRad Instrument. Reverse transcription was done using M-MuLV Reverse Transcriptase and Random Primer Mix (New England Biolabs Inc, USA). miRNA reverse transcription was performed using TaqMan miRNA reverse transcription reagents (Thermo Fisher Scientific, China) as per the instructions. Each sample was run in triplicate in 3 independent experiments.

### c-Myb transfection and silencing

Transfections were performed by transfecting cells with pcDNA3-c-myb containing the full-length cDNA for human c-myb, using Lipofectamine 3000 (Thermo Fisher Scientific, Shanghai, China). Cells were transfected with c-MYB-targeting siRNA or scrambled controls (Dharmacon, China), following the exact conditions as specified by manufacturer.

### Statistical analyses

Statistical analyses were done with Student’s *t* test. The level of significance was set at *p-*values less than <0.05 in all cases.

## Results

### Mesenchymal markers are up while miR-200s are down in tamoxifen resistant cells

**Figure 1:**
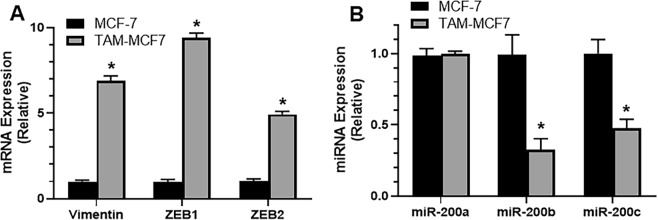
Parental MCF-7 cells and tamoxifen resistant MCF-7 cells (TAM-MCF7) were subjected to evaluation of EMT markers (**A**) and miR-200s (**B**), as indicated. Real time RT-PCR was performed with GAPDH as internal control. *p < 0.01.

With the intent to possibly find an EMT basis of tamoxifen resistance, and involvement of miR-200 family, we first evaluated the EMT markers such as vimentin, ZEB1 and ZEB2 for their mRNA expression, using real time RT-PCR. We used parental MCF-7 cells and compared the expression of markers in tamoxifen resistant TAM-MCF7 cells. As shown in Fig. 1A, the levels of all three markers, vimentin, ZEB1 and ZEB2 were elevated in TAM-MCF7 cells, as compared to the parental MCF-7 cells. Thus, we could verify that EMT is induced in our model of tamoxifen resistance.

We also evaluated the IC-50 values of tamoxifen in resistant cells vs. the parental ones and performed a dose-escalation experiment wherein cells were exposed to increasing concentrations of tamoxifen for different durations of time, and IC-50 of tamoxifen was calculated for the normal vs resistant cells. As shown in Table 1, whereas the IC-50 value decreased for tamoxifen, as would be expected for increasing time duration, the IC-50 values for tamoxifen resistant TAM-MCF7 cells were always higher than the parental MCF-7 cells at all the time points tested. In fact, the fold-changes in IC-50 values of TAM-MCF7 over IC-50 values of MCF-7 cells increased with the passage of time, which verified a robust resistance to tamoxifen in TAM-MCF7 cells (Table 1).

**Table 1:** Comparative time-dependent IC-50 values for tamoxifen in parental (MCF-7) and tamoxifen-resistance MCF-7 (TAM-MCF7) cells.

	MCF-7	TAM-MCF7	Fold Change^a^
*24 h*	9.86 ± 0.23	>50	
*48 h*	5.25 ± 0.19	36.74 ± 0.89	6.99
*72 h*	2.90 ± 0.17	21.32 ± 0.41	7.35
*96 h*	1.36 ± 0.10	14.23 ± 0.28	10.46

All values are in μM ± SE.

^a^Fold change in TAM-MCF7, compared to MCF-7 cells, calculated by TAM-MCF7/MCF-7.

With the goal of testing the possible involvement of miR-200s in tamoxifen resistance, we next evaluated the levels of all three miR-200s, miR-200a, miR-200b and miR-200c in the two paired cell lines. As shown in Fig. 1B, the levels of miR-200a were almost same in parental and resistant cells and there was no statistical significance. However, when we looked at the values for miR-200b and miR-200c, we could clearly see a significant (p < 0.001) change in their expression and their expression was markedly reduced in the resistant TAM-MCF7 cells, compared to parental MCF-7 cells. These results suggest a reduced expression of miR-200s, particularly miR-200a and miR-200c in our tamoxifen resistance model.

### miR-200b/c target c-MYB

**Figure 2:**
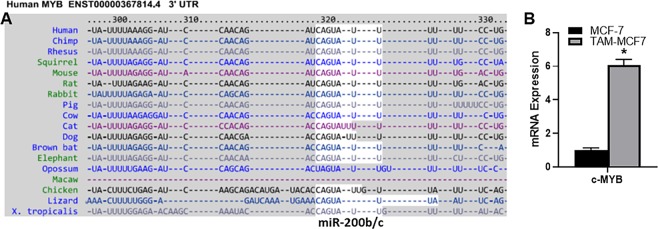
(**A**) TargetScanHuman 7.2 was used to list targets of miR-200s. c-MYB’s 3’UTR has conserved binding site for miR-200b/c. (**B**) c-MYB mRNA expression was assessed in parental MCF-7 cells and tamoxifen resistant MCF-7 cells (TAM-MCF7). Real time RT-PCR was performed with GAPDH as internal control. *p < 0.01.

Our next aim was to study functional role of miR-200b/c in tamoxifen resistance and that’s why looked into online databases to find a novel gene target of miR-200b/c. Using TargetScan (Release 7.2, 2018), we found c-MYB to be a possible target of both miR-200b and miR-200c (Fig. 2A). Since searches from online predictions need to be verified, we checked for c-MYB mRNA expression in our model and as shown in Fig. 2B, we found elevated c-MYB levels in TAM-MCF7 cells, compared to MCF-7 cells. These results suggest that c-MYB is a viable gene target of miR-200s and needs to be explored further.

**Figure 3:**
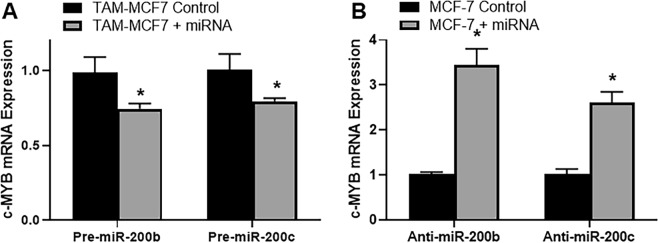
(**A**) Tamoxifen resistant MCF-7 cells (TAM-MCF7) were either transfected with control oligos or pre-miRNAs, as indicated, and then c-MYB expression was evaluated by real time RT-PCR, with GAPDH as internal control. (**B**) Parental MCF-7 cells were either transfected with control oligos or anti-miRNAs, as indicated, and then c-MYB expression was evaluated by real time RT-PCR, with GAPDH as internal control. *p < 0.01.

Further exploration came by way of studies that involved alterations in miR-200s’ expression followed by evaluation of c-MYB mRNA expression. First, we took TAM-MCF7, the resistant cells, and increased expression of miR-200s by transfections with pre-miR-200s. As shown in Fig. 3A, we observed significant, p < 0.01, decrease in c-MYB expression after transfections with both miR-200b and miR-200c. Verifying these results, when we put anti-miR-200b or miR-200c in MCF-7 parental cells, the levels of c-MYB shoot up. This indicates a direct regulation of c-MYB by miR-200s.

### c-MYB silencing reduces EMT and tamoxifen resistance

**Figure 4:**
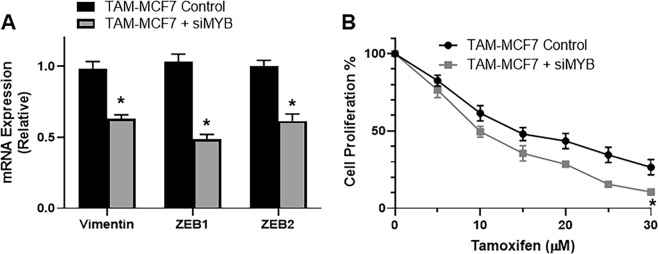
Tamoxifen resistant MCF-7 cells (TAM-MCF7) were either transfected with scrambled controls or c-MYB-specific siRNA and then either (**A**) evaluated for EMT markers, as indicated, by real time RT-PCR, with GAPDH as internal control or (**B**) treated with increasing concentrations of tamoxifen for 96 hours before MTS assay for proliferation. *p < 0.01.

If c-MYB expression is important for tamoxifen resistance and EMT is induced in tamoxifen resistance, there can be possible role of c-MYB in EMT induction. Therefore, we took resistant TAM-MCF7 cells and silenced c-MYB, by siRNA, and then evaluated EMT markers. As shown in Fig. 4A, all the three tested markers, vimentin, ZEB1 and ZEB2 were down-regulated in cells that were silenced for c-MYB. These results suggest that c-MYB is involved in EMT induction and since we already saw a correlation between EMT induction and tamoxifen resistance, we became interested in evaluating whether this observed reversal of EMT by silencing of c-MYB can also reverse the resistance to tamoxifen. For this, we treated TAM-MCF7 with si-c-MYB, and then exposed the cells to increasing concentrations of tamoxifen for 96 hours. As shown in Fig. 4B, we found a significant drop in resistance of TAM-MCF7 cells against tamoxifen. Also, as observed from the values provided in Table 2, the IC-50 value of siMYB-treated cells dropped to 9.92 μM from a value of 14.23 μM in control cells.

**Table 2:** IC-50 values for tamoxifen in cells with altered c-MYB expressions.

TAM-MCF7 Control	TAM-MCF7 – siMYB	T47D – Control	T47D + c-MYB
14.23 ± 0.28	9.92 ± 0.21	12.81 ± 0.19	19.14 ± 0.26

All values are in μM at 96 hours treatment with tamoxifen.

### c-MYB can similarly induce EMT and tamoxifen resistance in T47D cells

**Figure 5:**
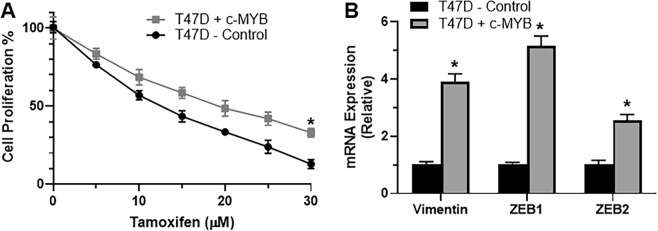
(**A**) T47D cells were either transfected with vector control or c-MYB-expressing cDNA and then either (**A**) treated with increasing concentrations of tamoxifen for 96 hours before MTS assay for proliferation or (**B**) evaluated for EMT markers, as indicated, by real time RT-PCR, with GAPDH as internal control. *p < 0.01.

Our results so far were in just one pared cell line that consisted of parental MCF-7 cells and the tamoxifen resistant TAM-MCF7 cells. To rule out cell line-specific effects, we used another cell line, T47D, which is also ER-positive. In these cells, we over-expressed c-MYB and evaluated tamoxifen resistance and EMT. As shown in Fig. 5A, c-MYB over-expression led to significantly increased resistance against tamoxifen, compared to the vector transfected T47D cells. This is evident from the values provided in Table 2. The IC-50 value of c-MYB overexpressing cells increased to 19.14 μM from a value of 12.81 μM in control cells. Further, all the mesenchymal markers (vimentin, ZEB1 and ZEB2) were significantly over-expressed in T47D cells transfected with c-MYB, compared to the control cells (Fig. 5B).

## Discussion

ER-positive breast cancers are blessed to have a targeted therapy but as seen with most targeted therapies, continued administration over a time period results in development of resistance. While human samples are precious and often difficult to obtain, cell line models are acceptable alternatives. In this study, we generated tamoxifen resistant breast cancer cells, MCF-7 and tried to understand the mechanisms of resistance, with the focus on EMT and the regulation by miRNAs, particularly those belonging to miR-200 family. The focus on miR-200s in this study was not without a logic. This family has a history of being implicated in resistance against multiple therapies in different cancers^15–17^; for example, EGFR targeting therapy in bladder cancer^18^ and lung cancer^19^, sorafenib and imatinib resistance in renal cancer^20^, oxaliplatin^21^ and 5-FU^22^ resistance in colon cancer, paclitaxel and carboplatin resistance in ovarian cancer^23^ and nintedanib^24^ and paclitaxel^25^ resistance in lung cancer. Despite the reports in several different cancers, there is very little evidence supporting such role of this miR family in tamoxifen resistance of breast cancers. Thus, our study fills that gap in the literature.

Having validated the down-regulation of miR-200s in our model system thus verifying a role of miR-200s in tamoxifen resistance, our primary task was to find a novel target gene that is regulated by miR-200s. We turned to bioinformatics which predicted c-MYB to be a potential target gene. c-MYB was predicted to be targeted by both miR-200b and miR-200c, and not by miR-200a. This is interesting because we found miR-200b and miR-200c to be significantly down-regulated in our model, and not the miR-200a. This, in itself, was a great evidence but we went ahead and validated the prediction our model system. Since miR-200b/c were downregulated, we expected c-MYB to be overexpressed. This is because miRNAs negatively regulate their target genes. We found this to be case as c-MYB was significantly upregulated in the tamoxifen resistant cells, compared to the parental cells.

Further to this evaluation of c-MYB in paired cell line model, we tested a direct regulation of c-MYB by miR-200b/c. Among the two cell lines in the paired cell lines, miR-200s were significantly downregulated in TAM-MCF-7 cells and therefore we tried to upregulate miR-200s in these cells by transfections with pre-miRs. When this was accomplished, we saw a significant downregulation of c-MYB as would be expected because of a negative regulatory relationship between miRNAs and its target. Furthermore, in the parental MCF-7 cells, where miR-200s were relatively at a higher endogenous levels, we tried to down-regulate miR-200s. This was accomplished by transfections with anti-miRs and resulted in significant upregulation of c-MYB as the negative regulation by miR-200s was now relieved. Thus, through multiple experiments, we clearly established a novel regulatory relationship between miR-200s and c-MYB.

Another important finding of this study is the role of miR-200-c-MYB in possibly regulating EMT which leads to tamoxifen resistance. The functional connection between c-MYB and EMT has been explored in several previous studies^26–29^ and moreover, the non-coding RNAs connection with EMT that involves c-MYB has also studied^30^. In an interesting earlier study^31^, it was reported that miR-200s such as miR-200b and miR-200c downregulate c-MYB in ER-positive cells and that EMT-inducing TGF-β stabilizes c-MYB. These results support our observation and may provide intricate details of miR-200 regulation of c-MYB. In our study, we did not find any effect of c-MYB on miR-200s (results not shown). We only report the regulation of c-MYB by miR-200s. However, there is evidence in the literature suggesting an activation of miR-200s by c-MYB^32^ which suggests that the relationship between miR-200s, c-MYB and EMT is complex and maybe even cell type or the cancer microenvironment dependent. More studies are needed to fully understand the mechanism.

## Data Availability

All data from this study is reported within the article.
